# Temperature-induced shifts in hibernation behavior in experimental amphibian populations

**DOI:** 10.1038/srep11580

**Published:** 2015-06-23

**Authors:** Xu Gao, Changnan Jin, Diego Llusia, Yiming Li

**Affiliations:** 1 Key Laboratory of Animal Ecology and Conservation Biology, Institute of Zoology, Chinese Academy of Sciences, 1 Beichen West Road, Chaoyang District, Beijing 100101, China; 2 University of Chinese Academy of Sciences, 19 Yuquan Road, Shijingshan District, Beijing 100049, China; 3 Chinese National Geography Magazine, jia 11, Datun Road, Chaoyang District, Beijing 100101, China; 4 Institut de Systématique, Évolution, Biodiversité, ISYEB UMR 7205 CNRS-MNHN-UPMC-EPHE, Muséum national d’Histoire naturelle, Sorbonne Universités, 57 rue Cuvier, CP 50, F-75005, Paris, France

## Abstract

Phenological shifts are primary responses of species to recent climate change. Such changes might lead to temporal mismatches in food webs and exacerbate species vulnerability. Yet insights into this phenomenon through experimental approaches are still scarce, especially in amphibians, which are particularly sensitive to changing thermal environments. Here, under controlled warming conditions, we report a critical, but poorly studied, life-cycle stage (i.e., hibernation) in frogs inhabiting subtropical latitudes. Using outdoor mesocosm experiments, we examined the effects of temperature (ambient vs. + ~2.2/2.4 °C of pre-/post-hibernation warming) and food availability (normal vs. 1/3 food) on the date of entrance into/emergence from hibernation in *Pelophylax nigromaculatus*. We found temperature was the major factor determining the hibernation period, which showed a significant shortening under experimental warming (6–8 days), with delays in autumn and advances in spring. Moreover, the timing of hibernation was not affected by food availability, whereas sex and, particularly, age were key factors in the species’ phenological responses. Specifically, male individuals emerged from hibernation earlier, while older individuals also entered and emerged from hibernation earlier. We believe that this study provides some of the first experimental evidence for the effect of climate warming on the timing of amphibian hibernation.

Recent climate change poses major challenges to the current biota. Global mean surface temperature has increased by approximately 0.8 °C over the last century and is likely to continue to increase 0.3–4.8 °C according to different emission scenarios for the 21st century^1^. Phenological shifts have been considered as one of the most pronounced climate change effects on biodiversity and as direct evidence of the influence of climate change on the life-history traits of species^2^,^3^,^4^,^5^. Examining such responses may contribute to a better understanding and the ability to predict species’ capacity to deal with climate change^6^. Yet current studies exploring phenological shifts in animals are mostly based on observational records, particularly in butterflies and birds^5^,^7^, and hence insights from experimental approaches are still scarce.

Mesocosm experiments have been widely used in climate-change research because they supply a suitable framework to establish casual relationships, weigh factor effects, and identify interactions and synergies (see review in^8^). Recently, temperature-induced phenological responses in insects and amphibians have been explored by experimental warming at the meso-scales^9^,^10^. Controlled warming elicited phenological mismatches in trophic interactions between Tibetan plants and insect herbivores^9^. Moreover, earlier metamorphosis in anuran larvae was reported under artificially warmed and dried environments, which allowed for an independent assessment of the factors involved^10^. We believe that our study is one of the first that experimentally examines the effect of climate warming on the timing of amphibian hibernation.

Recent studies on the impact of climate change on amphibians have focused on the timing of breeding^5^,^7^,^11^,^12^,^13^, especially in higher latitude areas^14^. While other key phenological events such as hibernation have mostly been neglected, despite sporadic studies on amphibian hibernation behavior from fieldwork were published^15^,^16^. Hibernation has evolved in many amphibians as protective mechanism in response to cold temperatures and, hence, plays a prominent role on amphibian life-history strategy by enabling survival under adverse environmental conditions^17^,^18^. However, markedly high temperatures during hibernation may also cause negative effects due to the increase in metabolic rate^19^, resulting in poor adult body condition after hibernation, declines in reproductive investment, and increase in mortality^20^,^21^,^22^. Moreover, some records suggest that climate warming also shortens the length of the hibernation period^5^,^21^. Such shortening might affect reproduction since hibernation has been shown to be a necessary life stage for the maturation of germ cells in some amphibians^23^.

Ecological impacts of climate change also include trophic mismatches. Phenological shifts produced by climate warming may affect secondary productivity and induce temporal mismatches alter trophic relationships within food webs^9^,^24^,^25^. Temperature-induced changes in phenology have led birds and other animals to mismatch peaks of breeding activity and food abundance^26^,^27^,^28^, which directly impacts their individual fitness and population dynamics. Similarly, shortening of hibernation periods might elicit trophic asynchrony between food resources and hibernating species. After emergence from hibernation, trophic mismatches might particularly hamper recovery of body condition, reduce or delay reproduction. In addition, changing food resources may also cause diverse effects on phenology, behavior, intra- and inter-specific relationships^7^,^21^,^24^. Thus, it is expected that an uncertainty in food availability favors more complex and less predictable responses of amphibians to climate change, such as the ones already observed in amphibian larvae showing differences in defensive strategies and growth patterns under warming conditions^10^,^29^,^30^, and further synergic processes related to phenological changes might emerge.

In this study, we experimentally examined the effects of temperature and food availability on the hibernation behavior of post-metamorphic black-spotted pond frogs (*Pelophylax nigromaculatus*) through outdoor mesocosm experiments. Specifically, we addressed the following questions: (i) Do frogs shift hibernation phenology when exposed to climate warming at rates consistent with projected climate-change scenarios? (ii) Is pre- and post-hibernation warming leading to similar shifting patterns? (iii) Does food deprivation cause synergic effects on species response to climate warming? and (iv) Do these responses differ across individual traits, such as sex or age? Using meso-scale experimentation with greenhouse units, we were able to test independent and interaction effects under controlled conditions, excluding other factors such as precipitation, population density, competitors, and predation pressure.

## Results

The timing of hibernation behavior of *P. nigromaculatus* was highly variable. The frogs entered into hibernation from day 294 to day 348 of 2011 (mean ± SD, 319.1 ± 13.9), and they emerged from hibernation between day 60 and day 105 of 2012 (mean ± SD, 74.9 ± 9.3). Selected sites for hibernation comprised underground holes and other refuges available in the mesocosm units. The differences in mean air temperature and the timing of hibernation across the experimental treatments are summarized in Table 1. The experimental population of each mesocosm was composed of a mean (±SD, range) of 8.6 (±0.5, 8–9) males and 5.8 (±0.4, 5–6) females. As revealed by skeletochronology, the age of the adult individuals averaged 2.3 years (±1.2, 1–6 years). The following three age classes were equally distributed among treatments: young (≤2 years, n = 163; one-way ANOVA, *F* = 1.786, *df* = 5, *P* = 0.119), adult (3–4 years, n = 79; *F* = 0.503, *df* = 5, *P* = 0.773) and old (≥5 years, n = 17; *F* = 0.712, *df* = 5, *P* = 0.627).

**Figure 1:**
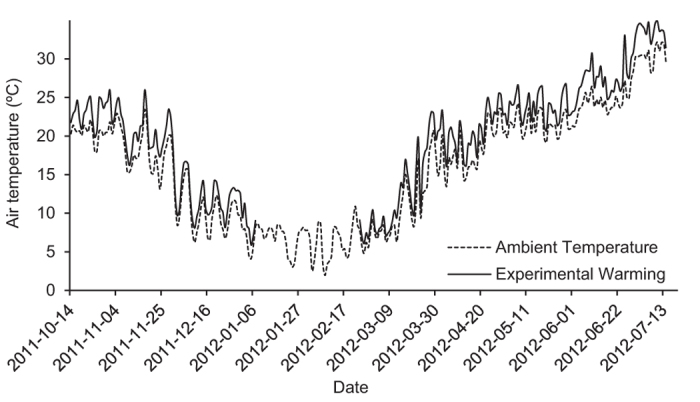
Air temperature (°C) recorded during the outdoor mesocosm experiments. Values correspond to daily average temperatures in 6 mesocosm units under ambient temperature and 12 mesocosm units exposed to experimental warming.

Air temperatures recorded in the mesocosms exposed to ambient temperature were on average 14.9 °C (4.1–23.4 °C) during the pre-hibernation period, 6.5 °C (2.0–10.9 °C) during hibernation and 19.5 °C (4.8–32.2 °C) during the post-hibernation period. Air temperatures recorded in the experimentally warmed mesocosms were on average 17.1 °C (5.9–26.0 °C) during the pre-hibernation period and 21.9 °C (6.1–35.0 °C) during the post-hibernation period (Fig. 1). Thereby, air temperatures in the warming treatments were on average 2.3 °C above these in other treatments: 2.2 °C (0.7–4.3 °C) for the pre-hibernation warming period and 2.4 °C (0.5–5.8 °C) for the post-hibernation warming period. Such an increase in air temperature is consistent with the predicted temperature rise (2.1–2.8 °C) in the experimental area by GISS-EH model A1B warming scenarios for the end of the century^31^, which reflect a medium long-term risk of global warming.

**Figure 2:**
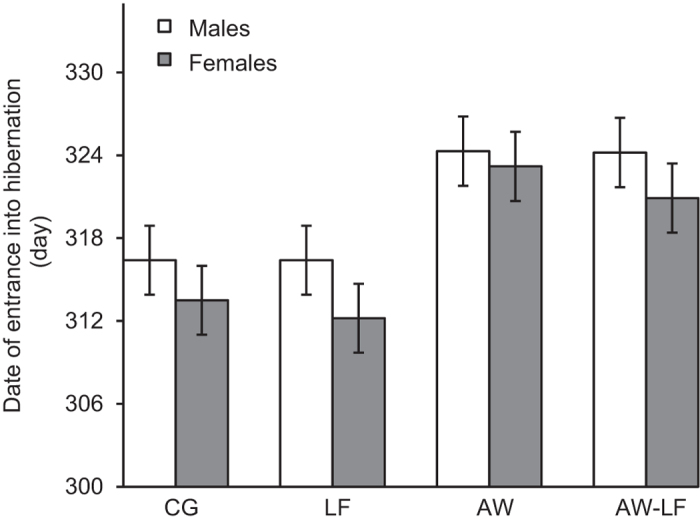
The average date of entrance into hibernation of *P. nigromaculatus* in outdoor mesocosms (mean ± SE) among experimental treatments: ambient temperature and normal (CG) or low food level (LF); and pre-hibernation warming (i.e., in autumn) and normal (AW) or low food level (AW-LF). The dates are day numbers starting from the 1^st^ of January 2011. The open bars indicate males, and the dark bars indicate females.

**Figure 3:**
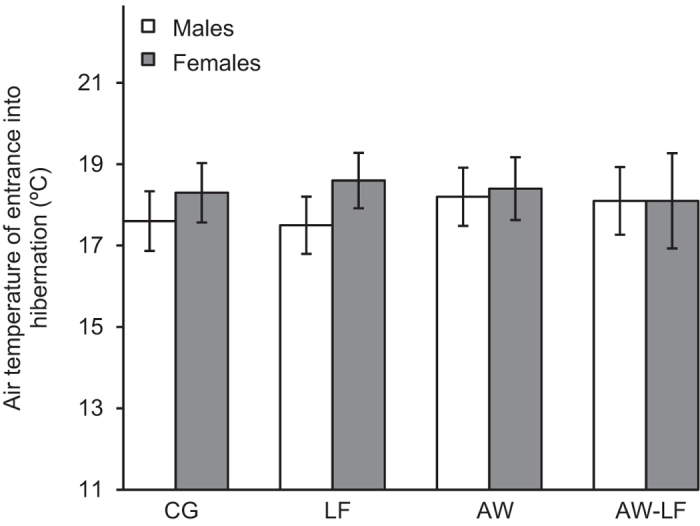
The average air temperature of entrance into hibernation (°C) of *P*. *nigromaculatus* in outdoor mesocosms (mean ± SE) among experimental treatments: ambient temperature and normal (CG) or low food level (LF); and pre-hibernation warming (i.e., in autumn) and normal (AW) or low food level (AW-LF). The open bars indicate males, and the dark bars indicate females.

The date of entrance into hibernation of *P. nigromaculatus* differed among experimental treatments, as shown by a mixed-model ANCOVA (Table 2). Pre-hibernation warming and the age of the subjects had a highly significant effect. Pairwise comparisons indicated that the experimental warming in autumn (pre-hibernation warming with normal food ‘AW’ or low food level ‘AW-LF’) delayed the date of entrance into hibernation relative to the exposure to ambient temperature (control group ‘CG’ and ambient temperature with low food level ‘LF’; *P* < 0.001; Fig. 2). The air temperature of entrance into hibernation was not significantly different among treatments (one-way ANOVA, *F* = 0.134, *df* = 3, *P* = 0.940; Fig. 3), and the air temperature was negatively correlated with the date of entrance into hibernation (Pearson correlation test, CG: *r* = −0.672, *P* < 0.001; LF: *r* = −0.675, *P* < 0.001; AW: *r* = −0.817, *P* < 0.001; AW-LF: *r* = −0.846, *P* < 0.001). The age of experimental subjects also showed a negative correlation with this phenological event (*β* = −0.011, *P* < 0.001). Older individuals entered hibernation earlier than young ones (Fig. S1). No significant difference in the entered into hibernation behavior was detected between individuals supplied with normal and low food levels or between sexes (food supply: *P* = 0.557; sex: *P* = 0.411; Fig. 2).

**Figure 4:**
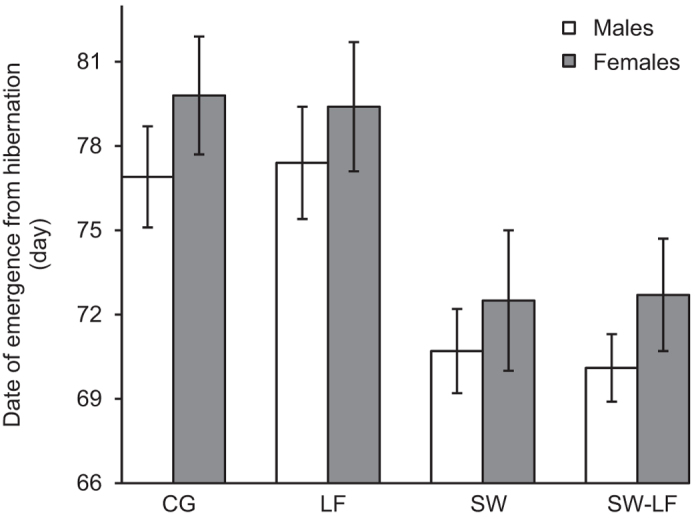
The average date of emergence from hibernation of *P*. *nigromaculatus* in mesocosm experiments (mean ± SE) among experimental treatments: ambient temperature and normal (CG) or low food level (LF); and post-hibernation warming (i.e., in spring) and normal (SW) or low food level (SW-LF). The dates are day numbers starting from the 1^st^ of January 2012. The open bars indicate males, and the dark bars indicate females.

**Figure 5:**
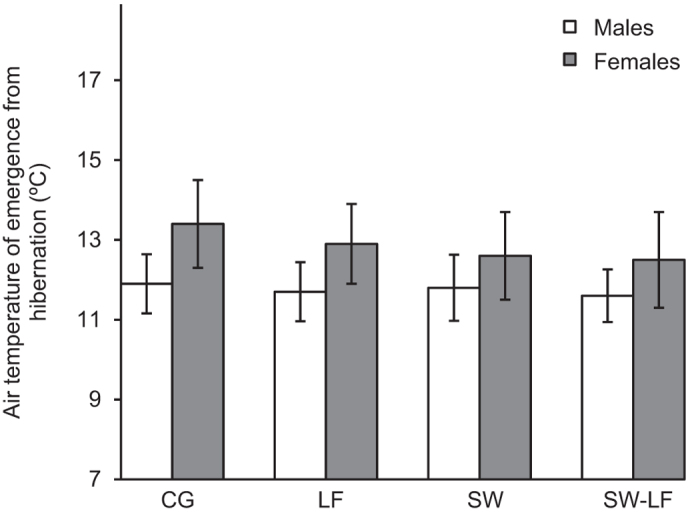
The average air temperature of emergence from hibernation (°C) of *P*. *nigromaculatus* in outdoor mesocosms (mean ± SE) among experimental treatments: ambient temperature and normal (CG) or low food level (LF); and post-hibernation warming (i.e., in spring) and normal (SW) or low food level (SW-LF). The open bars indicate males, and the dark bars indicate females.

The date of emergence from hibernation of *P. nigromaculatus* also differed among the experimental treatments (Table 2). Post-hibernation warming, the sex and age of the subjects had a highly significant effect. Pairwise comparisons indicated that the experimental warming in spring (post-hibernation warming with normal food ‘SW’ or low food level ‘SW-LF’) advanced the date of emergence from hibernation relative to the exposure to ambient temperature (CG and LF; *P* < 0.001; Fig. 4). The air temperature of emergence from hibernation was not significantly different among treatments (one-way ANOVA, *F* = 0.177, *df* = 3, *P* = 0.912; Fig. 5). As shown by a Pearson correlation test, air temperature of each treatment was positively correlated with this phenological event (CG: *r* = 0.828, *P* < 0.001; LF: *r* = 0.819, *P* < 0.001; SW: *r* = 0.901, *P* < 0.001; SW-LF: *r* = 0.831, *P* < 0.001). Moreover, pairwise comparisons indicated that males exhibited an earlier date of emergence from hibernation than females (*P* < 0.001; Fig. 4). The age of the experimental subjects showed a negative correlation with the date of emergence from hibernation (*β* = −0.030, *P* < 0.001). Older individuals emerged from hibernation earlier than young ones (Fig. S2). No significant difference in the emerged from hibernation behavior was detected between individuals supplied with normal and low food levels (*P* < 0.965; Fig. 4). Furthermore, the post-hibernation body size, weight, and body condition are affected by food available and original body parameters, while the post-hibernation body size and weight were also influenced by age (details are shown in Supplementary information Fig. S3-5 and Table S1). The original body condition (as a covariate) has no effect on the timing of hibernation of the species, as shown by the additional two ANCOVAs (Table S2).

## Discussion

Shifts in hibernation phenology were experimentally induced in outdoor mesocosm units in male and female of black-spotted pond frogs at both the entrance into and the emergence from this period of winter inactivity. (i) Temperature was the major environmental factor determining the hibernation period, which showed a significant shortening under experimental warming. (ii) Pre- and post-hibernation warming led to opposite shifting patterns, with delays in autumn and advances in spring, respectively, but of similar intensity (±6–8 days). In addition, (iii) the timing of hibernation was not affected by food availability, whereas (iv) sex and, particularly, age were key factors in the species’ phenological responses. Specifically, male individuals emerged from hibernation earlier, while older individuals also entered and emerged from hibernation earlier. The warming rate used to elicit shifts in hibernation phenology of the studied frogs was within the temperature range predicted by a moderate emission scenario for the end of this century (2.1–2.8 °C, GISS-EH model A1B warming scenarios)^31^. Thus, our results demonstrate that phenological patterns of hibernation in amphibians may be altered by climate warming as well as individual traits.

Temperature is a pervasive factor affecting most biological processes in amphibians^32^,^33^, and it is expected to be a prominent exogenous trigger of hibernation^6^,^7^. Here, experimental evidence for the effect of temperature on the phenology of amphibian hibernation was obtained from meso-scale experimentation. Both pre- and post-hibernation environmental temperature influenced the onset and duration of species activity. Our results are consistent with previous findings based on observational records in other anuran species^5^,^12^. The phenological shifts observed in this study may be caused by temperatures reaching the thermal thresholds of hibernation later (in autumn) or earlier (in spring) under experimental warming conditions. These findings suggest that current climate change might alter the hibernation behavior of the black-spotted pond frogs by shifting its phenological patterns. The biological consequences of such changes are unclear and warrant further study. Previous reports in other anurans found that phenological shifts in hibernation may affect energy reserves and body condition during and after hibernation, with potential implications on individual fitness and survival^20^,^21^,^34^. Furthermore, phenological shifts might also be involved in changes in ecological interactions, such as temporal mismatches in trophic synchrony^25^.

The effects of climate change are expected to be particularly severe in amphibians, which are physiologically restricted in their ability to control body temperature and exceedingly sensitive to changes in both temperature and humidity^35^,^36^. Therefore, amphibians have shown trends in delaying or advancing phenophases as observed in other organisms^37^,^38^, but with more diverse pattern shifts and a significantly stronger intensity^2^,^5^,^7^,^12^. Recent studies document that amphibians at higher latitudes are experiencing higher increases in temperature and therefore exhibit greater phenological shifts in response to climate change^14^. These results might suggest that more pronounced shifts to later hibernation would be found in temperate zone amphibians than in the amphibians that inhabiting subtropical latitudes, such as the species in this study. In addition to phenological shifts, changing thermal environments also affect a species’ life-history strategies and risk of extinction and cause changes in its distribution patterns, abundance, body size and interspecific interactions^3^,^4^,^39^,^40^,^41^,^42^.

As an indirect effect of climate change, food availability and predictability may be altered by phenological mismatches in food webs^24^ and this, in turn, might exacerbate changes in animal behavior. However, food availability did not influence the entrance or emergence date of hibernation in the study species. Despite of a reduction to one-third of the normal food availability, the black-spotted pond frogs did not show shifts in the timing of hibernation behavior in comparison with those in normal food supply treatments. This finding contrasts with experimental records in mammals such as arctic ground squirrels (*Urocitellus parryii*), which show a shorter hibernation period associated with increases of food availability^43^. These different responses may be due to the physiological mechanisms characterizing endotherms and ectotherms.

Our results suggest that hibernation behavior of the black-spotted pond frogs might have a sort of tolerance to short-term food deprivation during pre-hibernation periods, enabling them to cope with food unpredictability and changing sources. Nevertheless, the observed lack of relationship between food availability and hibernation phenology might also be due to deprivation conditions that were not intense enough in rate or time (e.g., overrated food supplies, short-term experimental condition, etc.). However, food deprivation may affect animal traits other than phenology. Shifts in food availability have been found to strongly influence energy reserves and overwintering body conditions in the common toad (*Bufo bufo*), and, thereby, mortality rate during hibernation^21^. Available evidence suggests that organism at higher trophic levels respond to changing environments at different rates compared to those from lower trophic levels^26^,^27^. As predators, amphibians are therefore exposed to a risk of trophic level asynchrony that may exacerbate their vulnerability to climate change^44^. Further investigations should examine these synergic processes that determine and are influenced by hibernation phenology.

Individual traits were also found to be relevant factors affecting the timing of hibernation behavior, differing between males and females and among age classes. While both sexes entered into hibernation at similar dates, males emerged from hibernation earlier than females. Such differences in the phenology of spring events is probably associated with the different sexual roles played by individuals of each sex during the reproductive season, when males are the first to occupy the breeding points in permanent water bodies^45^. Similar cases also occur in other species or taxa^43^,^46^. Moreover, the age of the subjects caused advancement in both the entrance into and emergence from hibernation. As adult amphibians tend to invest a greater proportion of energy in reproduction than younger ones^47^, the difference in hibernation phenology related to age seems to be caused by different growth and reproduction allocation strategies. Adult females of arctic ground squirrels (*U. parryii*) were observed entering into hibernation earlier than juveniles, which was also related to their reproduction strategy^43^. The individual experience offers another explanation for the differences in hibernation phenology among age classes^48^. As it is expected that adult individuals find hibernation sites more efficiently than young ones, age and individual experience might influence the timing of hibernation, although such hypothesis should be further tested. Interactions between these individual traits and environmental factors such as temperature and food supply were not observed in the phenological responses of black-spotted pond frogs.

This study, together with other recent works^8^, stresses the significance of meso-scale experimentation in ecological climate-change research. This approach may provide experimental support for observed trends and establish cause-effect relationships in animal behavioral responses to changing environments. Future investigations should further explore such a research framework, particularly with environmental factors other than temperature, such as precipitation, in order to disentangle the complex mechanisms that underlie a species’ phenological patterns.

## Materials and Methods

### Ethics Statement

This study was conducted under the approval of the Animal Care and Ethics Committee, Institute of Zoology, Chinese Academy of Sciences (Project No. 2011/43). The permit to collect animals for use in the mesocosm experiments was obtained from the Forestry and Environmental Protection Departments of the town of Taohua. The frog in this study was not an endangered or protected species. All staff in this study received appropriate training before performing the animal studies. The experiments were carried out in accordance with the guidelines for the Use of Animals in Research issued by the Institute of Zoology, Chinese Academy of Sciences.

### Study site

Outdoor mesocosm experiments were performed on Taohua Island, Zhejiang, China (29.85° N, 122.26° E) from 14 October 2011 to 14 July 2012. The site is an agricultural ecosystem comprising farm fields, grasslands (*Eleusine indica*, *Echinochloa* spp. and *Arundo* spp.), and several ditches and ponds. It has a humid subtropical climate regimen (*Cfa*) under the Köppen climate classification^49^, is highly seasonal, and has an annual precipitation of 936.3–1330.2 mm and has a mean annual temperature of 15.6–16.6 °C; January is the coldest month (mean temperature approximately 5 °C) and August is the hottest month (mean temperature approximately 27 °C)^50^.

### Study species

The black-spotted pond frog *P. nigromaculatus* (Hallowell, 1861) is a large-sized anuran species (maximum snout-vent length: 70 mm for males and 90 mm for females) that is widely distributed throughout most of China, far eastern Russian, Japan, and the Korean peninsula^51^,^52^. This ranid feeds primarily on insects, crustaceans, arachnids, and gastropods^53^. The species breeds after hibernation, usually from late March to early July in the study area^54^. Hibernation takes place underground, mainly in burrows, ground holes, under fallen leaves or small branches, and in other frost-proof refuges^52^. This frog is one of the most common and abundant amphibian species in mainland China, often found in agroecosystems, water bodies, and surrounding areas, allowing adequate sample sizes for experimental purposes^52^.

### Experimental procedures

**Figure 6:**
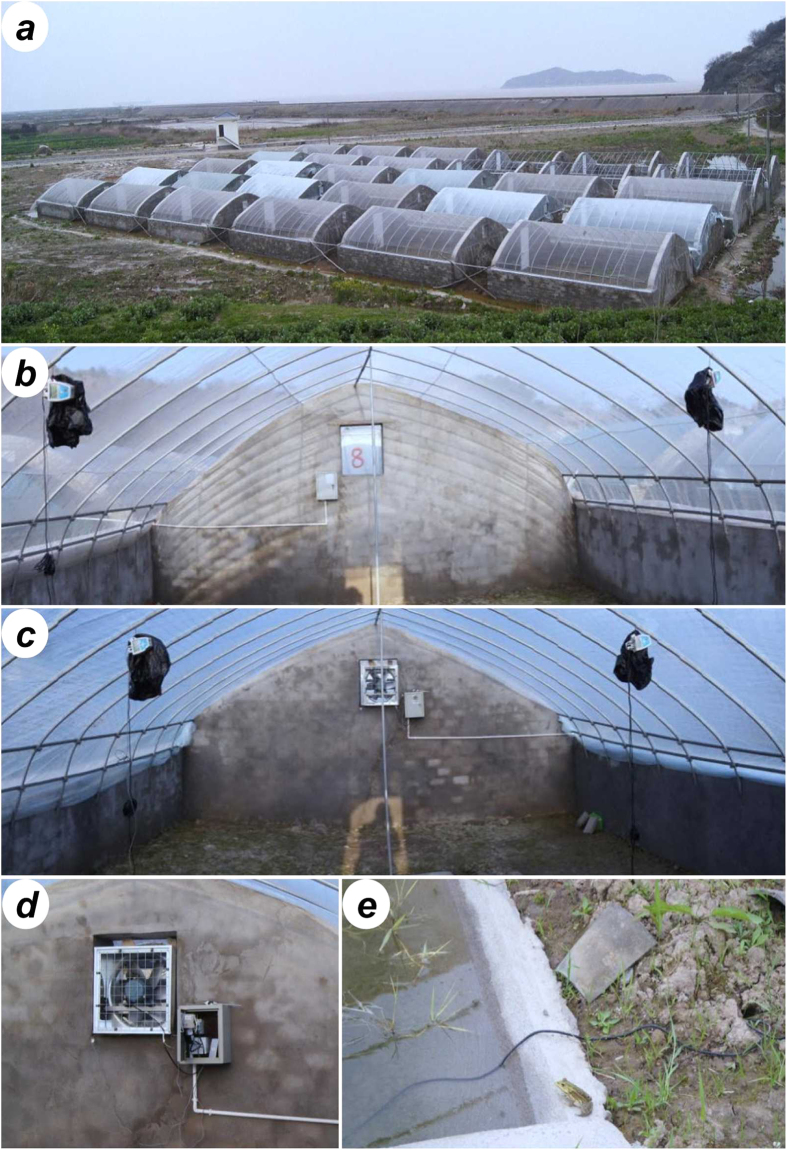
The mesocosm experiment photos. (**a**) The mesocosm units in the field; (**b**) The ambient temperature mesocosm unit; (**c**) The experimental warming mesocosm unit; (**d**) The real-time temperature monitoring equipment; (**e**) A black-spotted pond frog staying at the edge of a pond in a mesocosm unit. Photo credits: Xu Gao.

Experiments were carried out in 18 mesocosm units. Each unit consisted of a 7 × 6.5 m grassland area, surrounded by 1.2 m tall brick walls, and with a 3 × 1.2 m pond (0.5 m deep) in the middle of the mesocosm. These units were 1 m apart from the walls of the closest unit. The soil in mesocosm units was soft and designed with refuges to facilitate access of the species to hibernation sites. An arc-shaped steel frame for greenhouse (2.2 m at the top) was installed in each unit to provide a cover over the entire mesocosm area during the experimental treatments (Fig. 6).

To examine the effect of temperature and food supply on the timing of amphibian hibernation, a 3 × 2 factorial experiment was designed as follows. An experimental design with two independent warming periods (pre- and post-hibernation) was employed to identify the specific contribution of thermal conditions of each period in the hibernation phenology of the study species. It should be noted that the use of independent periods of experimental warming might underestimate the intensity of the phenological shifts for individuals subjected to post-hibernation treatment and, hence, not subjected to the warming treatment for the entire hibernation period. Frogs in the mesocosm units were exposed to one of six treatments, composed of a combination of three distinct thermal conditions (ambient temperature, pre-hibernation warming, or post-hibernation warming) and two levels of food supply (normal or low). The control group (CG) experienced ambient temperature and was supplied with a normal level of food. The treatment groups were exposed to the following: (i) ambient temperature with low food level (LF); (ii-iii) pre-hibernation warming (i.e., in autumn) with normal (AW) or low food level (AW-LF); (iv-v) post-hibernation warming (i.e., in spring) with normal (SW) or low food level (SW-LF). Each of these six treatments had three replicates that were randomly assigned to the mesocosm units.

Experimental warming at both pre- and post-hibernation periods was conducted through the greenhouse method. The mesocosm tops were covered with ethylene vinyl acetate films (EVA, thickness: 0.1 mm, Xifeng Co., Ltd., Baishan, China), containing holes (diameter: 25 mm, 8 holes per square meter) for permeability to precipitation. The amount of rainfall measured throughout the study was not significantly different between the experimental warming and ambient temperature treatments (one-way ANOVA, *F* = 0.055, *df* = 1, *P* = 0.815). Air humidity was on average 81.3 ± 8.1% (mean ± SD) in the warmed mesocosms, while 79.6 ± 9.1% in the unwarmed mesocosms. Pre-hibernation warming (treatments AW and AW-LF) lasted from the onset of the experiments to the entrance into hibernation of all the frogs (85 days). Analogously, post-hibernation warming (treatments SW and SW-LF) lasted from the late hibernation period to the end of the experiments (141 days). Specifically, the post-hibernation warming started on the 24^th^ of February 2012, after the first three days of hibernation with daily mean temperature above 8 °C, which is considered to be a critical temperature for some species^55^. During the remaining experimental period, greenhouse coverings were changed to insect screens (40 mesh, Jinnong Mesh Factory, Taizhou, China) so that subjects experienced ambient temperatures. In the other treatments (CG and LF), mesocosm units were also covered with insect screens during the entire experimental period.

To control the temperature regime and simulate a daily fluctuating temperature in the mesocosm units, air temperature of the warming treatments was automatically regulated by real-time temperature monitoring equipment assembled ad hoc (Fig. 6d). The equipment consists of a louvered exhaust fan (CY-4G, Chengyi Environmental Protection Device Co., Ltd., Dongguan, China) and a temperature difference controller (LC-215B+, Besful Electric Co., Ltd., Shenzhen, China) that measured air temperature in both the warming treatments and the control treatments. When air temperature in the experimental warming treatments was 4 °C above air temperature in the control treatments, the temperature difference controller activated the exhaust fan to expel warmed air and to reduce the temperature difference between mesocosms to 1 °C. Then, the temperature difference controller closed the fan until the temperature again exceeded the 4 °C difference. The noise produced by the exhaust fan reached sound pressure levels similar to those of the background noise in the study area (approximately 56 dB); hence, we assumed it had a negligible effect on frog behavior. During the experiments, the air temperature in each mesocosm was recorded hourly with environmental data loggers (RC-500+, Jingchuang Electric Co., Ltd., Xuzhou, China). In addition, we also measured the soil temperature, which was defined as the temperature of surrounding soil in a typical hideout of the frogs^15^. Air and soil temperature were highly correlated in these treatments (Pearson correlation test, ambient temperature: *r* = 0.916, *P* < 0.001; experimental warming: *r* = 0.887, *P* < 0.001; Fig. S6).

The food supply for the experimental subjects consisted of commercial crickets (*Gryllus bimaculatus*, approximately 15 mm in length) dusted with calcium powder. In the treatments of normal food level, each frog was fed with crickets every night according to its body mass at the onset of the experiments. The feeding ratio was 1 cricket for every 20 g of frog body mass, with the subjects being classified into four levels^56^: ≤20 g of body mass (n = 117), 20–40 g (n = 124), 41–60 g (n = 21), and >60 g (n = 8). In generally the frogs preyed on the crickets immediately, so that 1 min per frog at most was assigned as feeding time. After that, those crickets that were not eaten were removed from the mesocosm units. In the treatments of low food level, the frogs were fed every 3 days (i.e., equaling 1/3 of the normal food supply^57^).

In total, 270 post-metamorphic frogs were captured from the wild in Maoshan Village in the town of Taohua (29.49°N, 122.15°E) in mid-October 2011. Fifteen captured frogs were randomly assigned to each mesocosm. Before the start of the experiments, PIT-tags (2 × 8 mm, HT950, HongTeng Barcode Technology Co., Ltd, Guangzhou, China) were injected into the muscles of the right thigh of each subjects. Each tag contains a unique identification code so that all the marked frogs could be individually identified with a hand-held scanner (KD-Pi60, Kingdoes RFID Technologies Co., Ltd, Beijing, China). The code of each individual was recorded every day until its entrance into hibernation and then from its emergence from hibernation to the end of experiments. During the study, a total of 11 subjects died across the different mesocosm units and they were excluded from the data analysis. Immediately after the experimental treatments, frogs were toe-clipped and their ages were subsequently estimated by skeletochronology^58^. Assuming that all the experimental subjects had a skeletal age of 1 year or older at the time of toe clipping, the sex was determined based on the presence of secondary sexual characteristics. Frogs lacking such characteristics were identified as females^52^.

### Statistical analyses

To determine the relationship between air temperature and the date of entrance into and emergence from hibernation, we used Pearson correlation analyses. Differences in the date of entrance into and emergence from hibernation across the experimental treatments were examined using a mixed model ANCOVA with sex, temperature and food supply as the fixed effects and the replicates of each treatment as the random effects. Because the age of the individuals might affect their hibernation behavior, this factor was also included in the statistical tests as a covariate. Frogs may temporarily enter a dormant phase before and after hibernation, and hence we used the date of their last and first appearances in the mesocosm as the dependent variables. These variables were defined as a day number throughout the year^40^, from the 1^st^ of January (=1) to the 31^th^ of December (=365). We logarithmically transformed the date of entrance into and emergence from hibernation to minimize the heterogeneity of variances^59^. All analyses were conducted using R v2.15.3 (R Development Core Team 2012). Normality and homoscedasticity of the model residuals were determined with Kolmogorov-Smirnov tests and Levene's tests. Results were considered significant if *P* ≤ 0.05 (*α* ≤ 0.05).

## Additional Information

**How to cite this article**: Gao, X. *et al.* Temperature-induced shifts in hibernation behavior in experimental amphibian populations. *Sci. Rep.* **5**, 11580; doi: 10.1038/srep11580 (2015).

## Supplementary Material

Supplementary Information

## Figures and Tables

**Table 1:** Mean air temperature and the timing of entrance into/emergence from hibernation of *P.*

**Treatment**	**Entrance into hibernation**		**Emergence from hibernation**	
	**Mean air Temperature (°C)**	**Mean date (2011)**	**Mean air Temperature (°C)**	**Mean date (2012)**
CG	17.9 ± 3.4	315.0 ± 12.5	12.3 ± 4.0	78.1 ± 9.0
	(8.5–22.9)	(294–336)	(6.7–20.7)	(63–100)
LF	17.9 ± 3.4	314.7 ± 12.3	11.9 ± 4.0	78.0 ± 9.8
	(8.5–22.9)	(294–337)	(6.3–20.7)	(63–105)
AW	18.4 ± 3.5	323.1 ± 14.3	-	-
	(9.6–23.6)	(297–348)	-	-
AW-LF	18.1 ± 4.5	322.9 ± 14.4	-	-
	(9.1–23.6)	(299–348)	-	-
SW	-	-	12.2 ± 4.3	71.6 ± 8.9
			(7.4–23.4)	(60–93)
SW-LF	-	-	11.8 ± 4.0	71.8 ± 7.2
			(7.9–23.2)	(61–89)

***Nigromaculatus*** **(mean ± SD, range) among experimental treatments**: ambient temperature and normal (CG) or low food level (LF); pre-hibernation warming (i.e., in autumn) and normal (AW) or low food level (AW-LF); and post-hibernation warming (i.e., in spring) and normal (SW) or low food level (SW-LF).

**Table 2:** Summary of two mixed-model ANCOVAs for the date of entrance into/emergence from hibernation of *P. nigromaculatus* in outdoor mesocosm experiments, with experimental warming, food supply, and sex as main effects, replicates of each treatment as a random variable, and age as a covariate.

**Source of variation**	**Date of entrance into hibernation (log**_**10**_**- transformed)**		**Date of emergence from hibernation**	
	***df***	***F***	***df***	***F***
Pre-hibernation warming	1	43.760***	-	-
Post-hibernation warming	-	-	1	52.769 ***
Food supply	1	0.347	1	0.002
Sex	1	0.679	1	16.894***
Age	1	240.202***	1	175.264***
Replicates of each treatment	2	1.321	2	0.153
Pre-hibernation warming × Food supply	1	0.295	-	-
Pre-hibernation warming × Sex	1	0.748	-	-
Pre-hibernation warming × Food supply × Sex	1	<0.001	-	-
Food supply × Sex	1	0.865	1	0.013
Post-hibernation warming × Food supply	-	-	1	0.025
Post-hibernation warming × Sex	-	-	1	0.035
Post-hibernation warming × Food supply × Sex	-	-	1	0.215
Error	163		159	

All interaction terms are also considered.

*P*-value < 0.001 (2-tailed).
