# Supplementary material for: Temperature-induced shifts in hibernation behavior in experimental amphibian populations
Source: Sci Rep. 2015 Jun 23;5:11580. doi: 10.1038/srep11580 (PMC4477341; doi:10.1038/srep11580)
Supplement: Supplementary Information [file srep11580-s1.pdf]

Scientific Reports

Supplementary Information

Temperature-induced shifts in hibernation behavior in experimental amphibian populations

Xu Gao[1, 2†], Changnan Jin[1, 2, 3†], Diego Llusia[4] and Yiming Li[1*]

[1] *Key Laboratory of Animal Ecology and Conservation Biology, Institute of Zoology, Chinese Academy of Sciences, 1 Beichen West Road, Chaoyang District, Beijing 100101, China;* [2] *University of Chinese Academy of Sciences, 19 Yuquan Road, Shijingshan District, Beijing 100049, China;* [3] *Chinese National Geography Magazine, jia 11, Datun Road, Chaoyang District, Beijing 100101, China;* [4] *Institut de Systématique, Évolution, Biodiversité, ISYEB UMR 7205 CNRS-MNHN-UPMC-EPHE, Muséum national d'Histoire naturelle, Sorbonne Universités, 57 rue Cuvier, CP 50, F-75005, Paris, France.*

Correspondence and requests for materials should be addressed to Y.L. (liym@ioz.ac.cn);

†Co-first authors contributed equally to this work.

This Supplementary Information contains 6 figures and 2 tables.

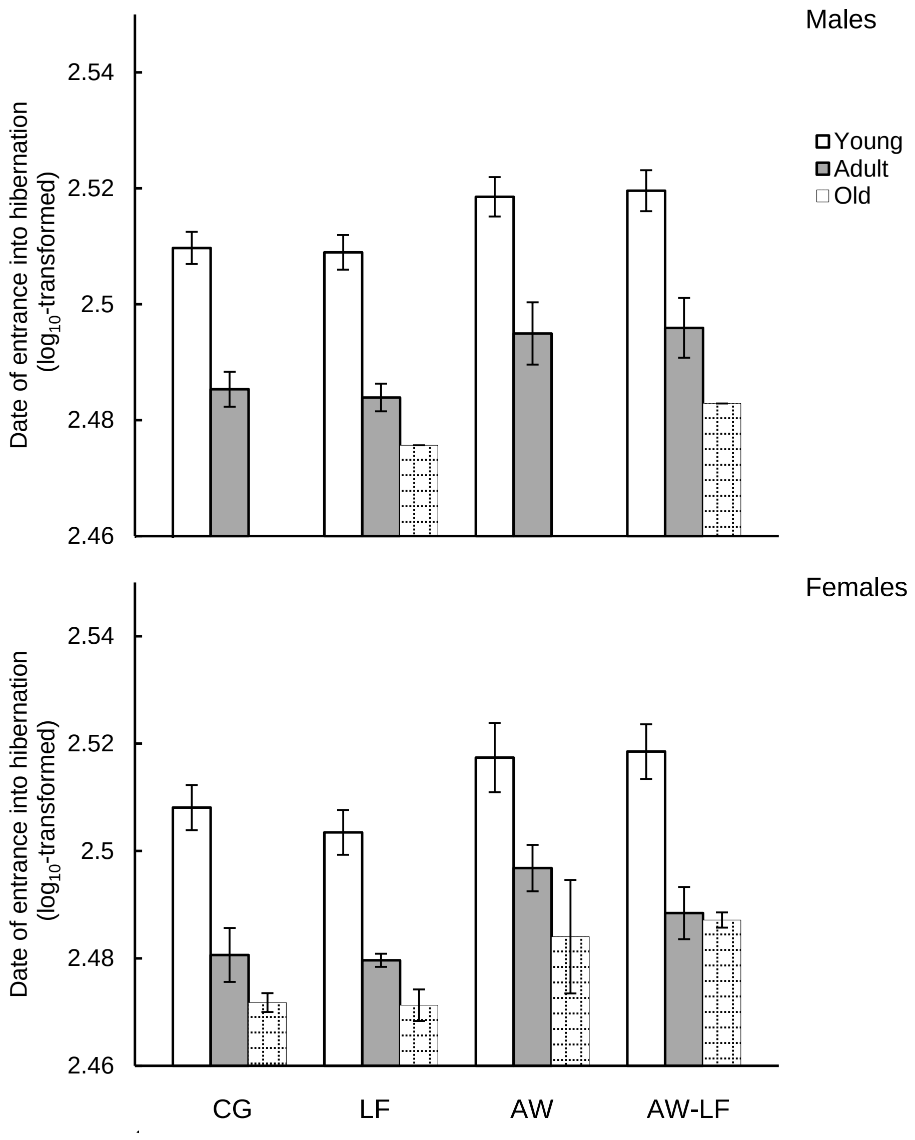

Figure S1 The average date of entrance into hibernation of different age classes of *P. nigromaculatus* among experimental treatments: ambient temperature and normal (CG) or low food level (LF); and pre-hibernation warming (i.e., in autumn) and normal (AW) or low food level (AW-LF). Open bars indicate young (≤ 2 years), dark bars indicate adult (3 – 4 years), and grid pattern bars indicate old (≥ 5 years).

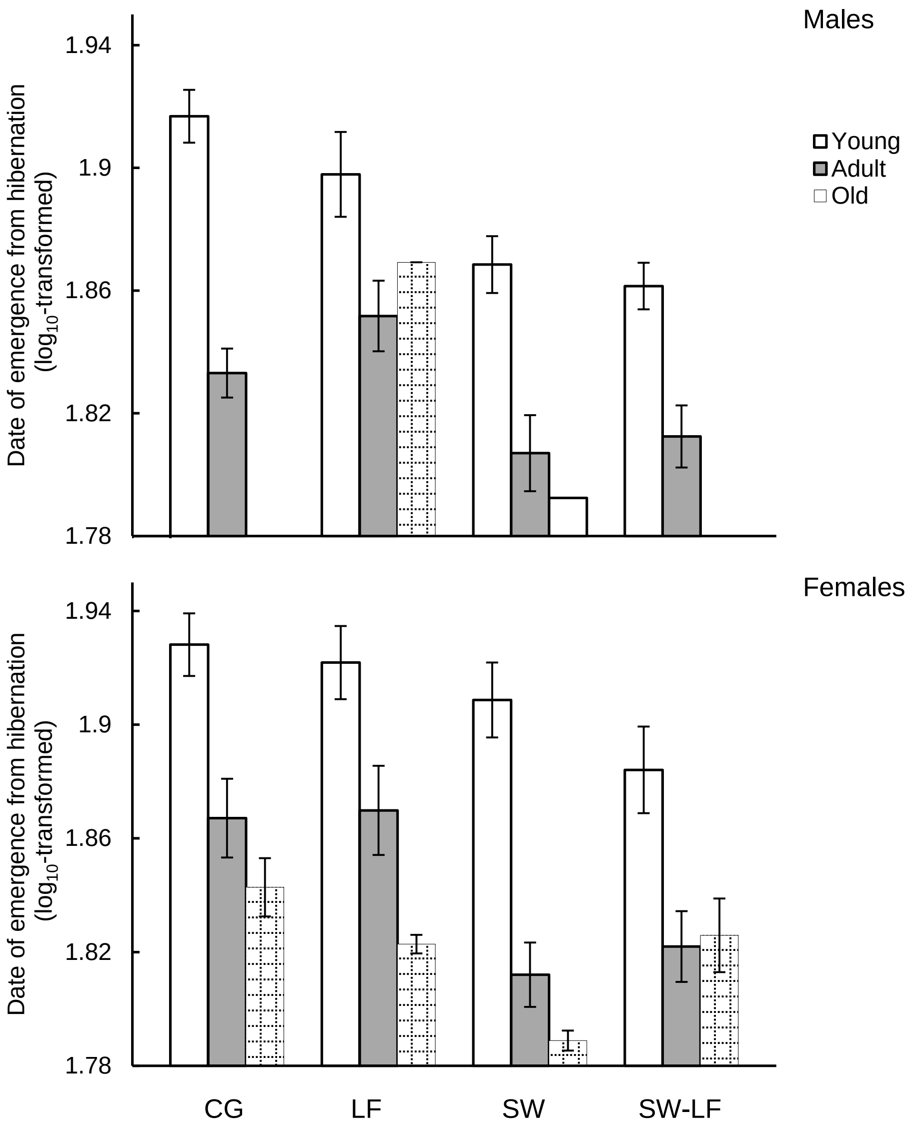

Figure S2 The average date of emergence from hibernation of different age classes of *P. nigromaculatus* among experimental treatments: ambient temperature and normal (CG) or low food level (LF); and post-hibernation warming (i.e., in spring) and normal (SW) or low food level (SW-LF). Open bars indicate young (≤ 2 years), dark bars indicate adult (3 – 4 years), and grid pattern bars indicate old (≥ 5 years).

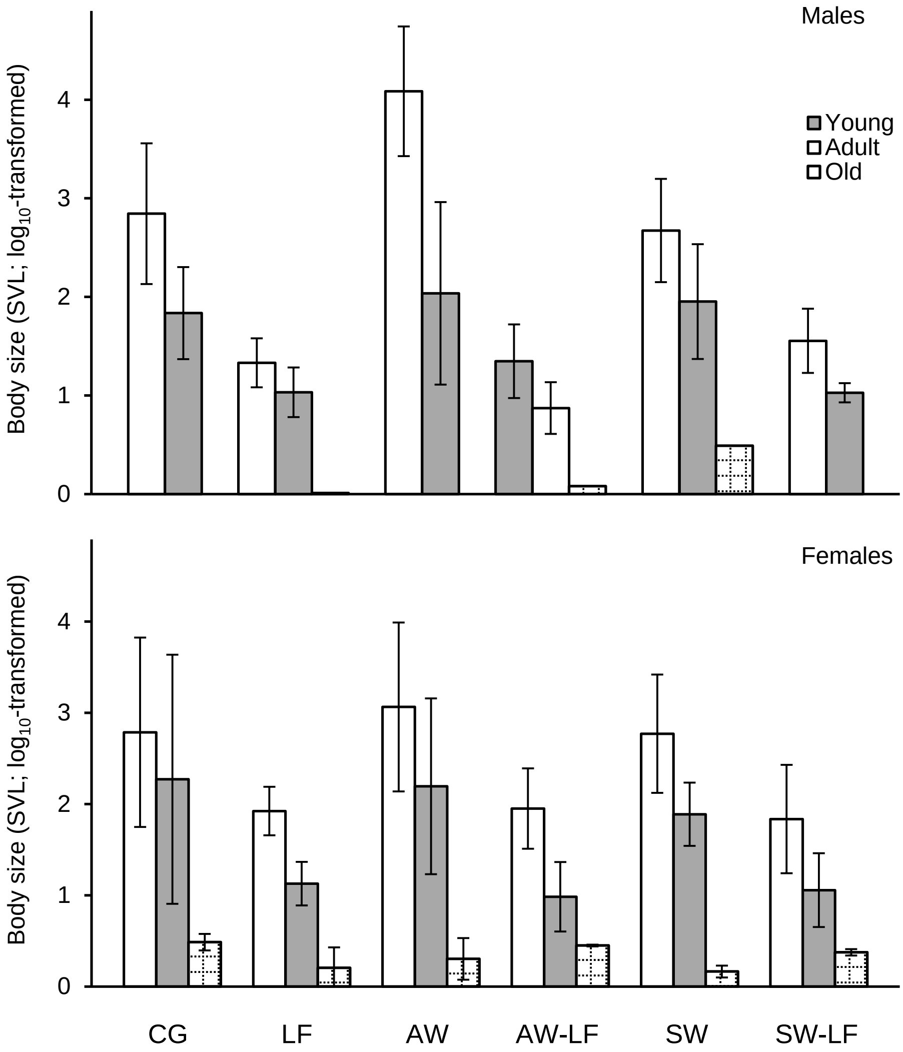

Figure S3 The variation of body size (SVL) after hibernation of different age classes of *P. nigromaculatus* among experimental treatments: ambient temperature and normal (CG) or low food level (LF); pre-hibernation warming (i.e., in autumn) and normal (AW) or low food level (AW-LF); and post-hibernation warming (i.e., in spring) and normal (SW) or low food level (SW-LF). Open bars indicate young (≤ 2 years), dark bars indicate adult (3 – 4 years), and grid pattern bars indicate old (≥ 5 years).

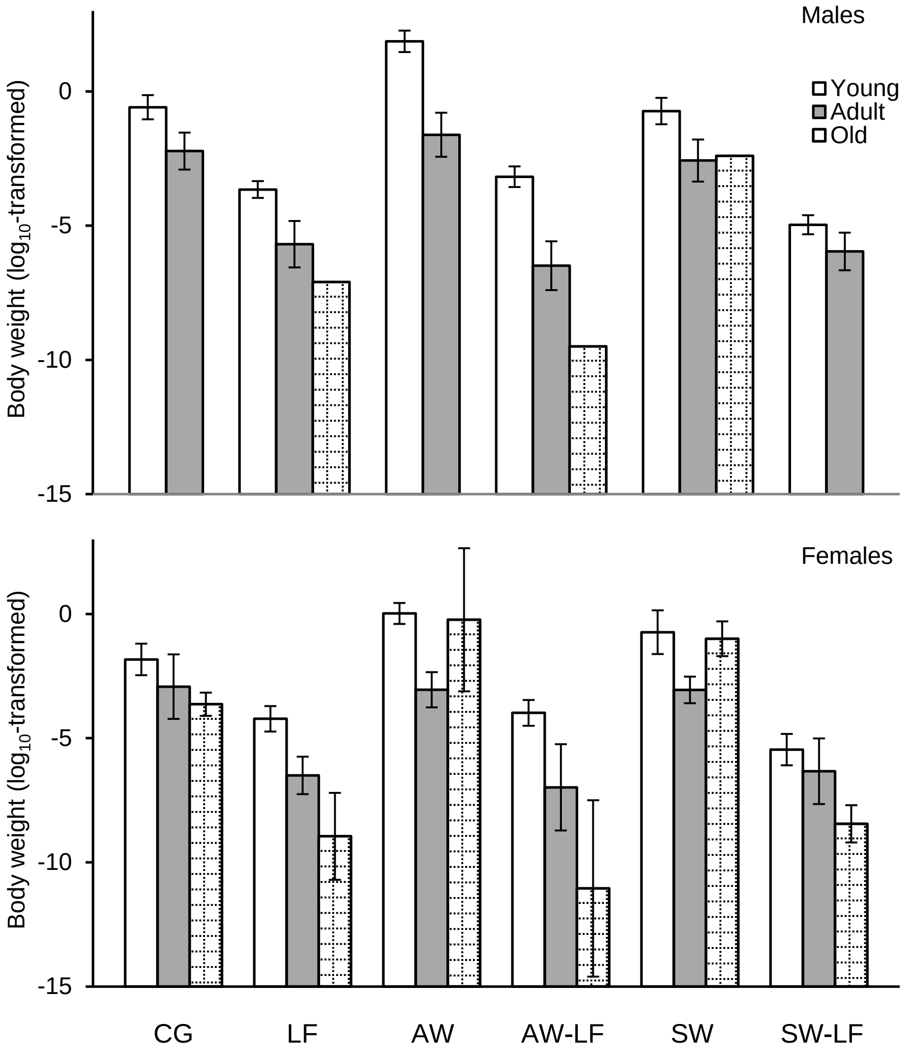

Figure S4 The variation of body weight after hibernation of different age classes of *P. nigromaculatus* among experimental treatments: ambient temperature and normal (CG) or low food level (LF); pre-hibernation warming (i.e., in autumn) and normal (AW) or low food level (AW-LF); and post-hibernation warming (i.e., in spring) and normal (SW) or low food level (SW-LF). Open bars indicate young (≤ 2 years), dark bars indicate adult (3 – 4 years), and grid pattern bars indicate old (≥ 5 years).

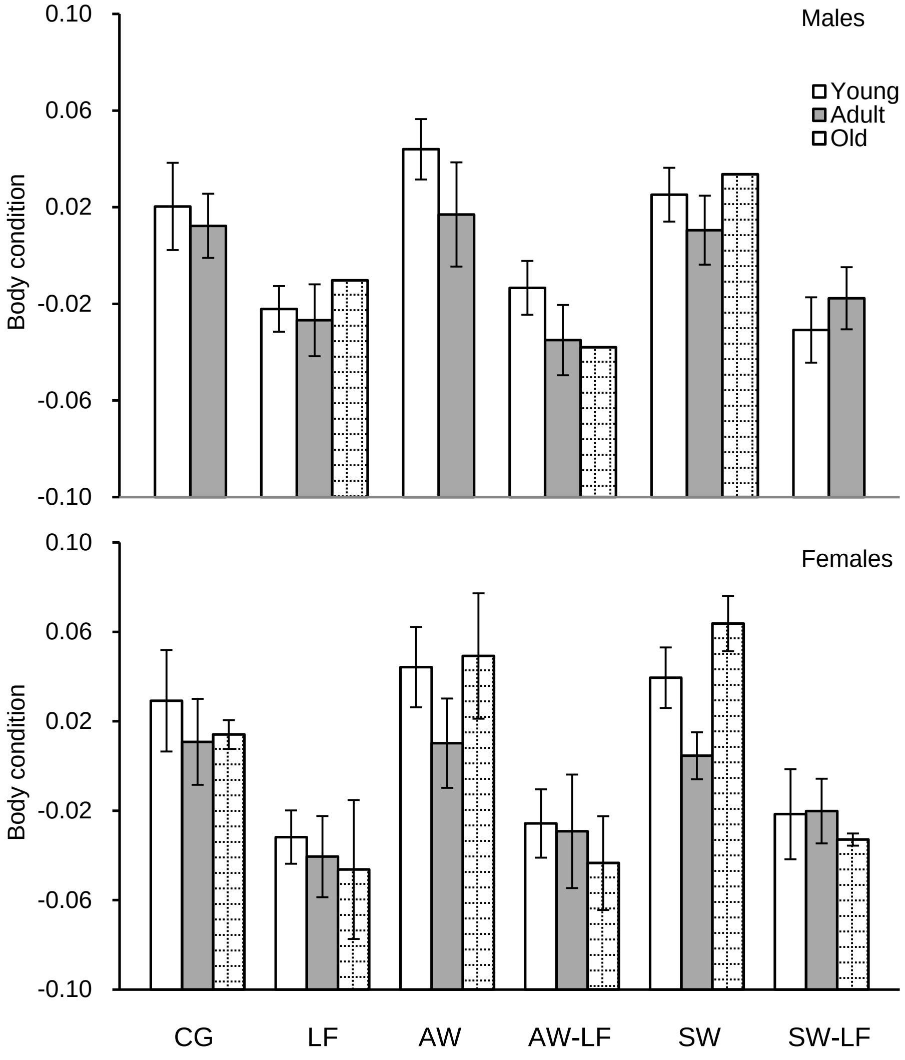

Figure S5 The variation of body condition after hibernation of different age classes *P. nigromaculatus* among experimental treatments: ambient temperature and normal (CG) or low food level (LF); pre-hibernation warming (i.e., in autumn) and normal (AW) or low food level (AW-LF); and post-hibernation warming (i.e., in spring) and normal (SW) or low food level (SW-LF). Body condition was determined as the residual of the regression analysis of $\log_{10}$ weight against $\log_{10}$ SVL[1], each sex calculated respectively.

1. Reading, C. J. Linking global warming to amphibian declines through its effects on female body condition and survivorship. *Oecologia* **151**, 125-131 (2007).

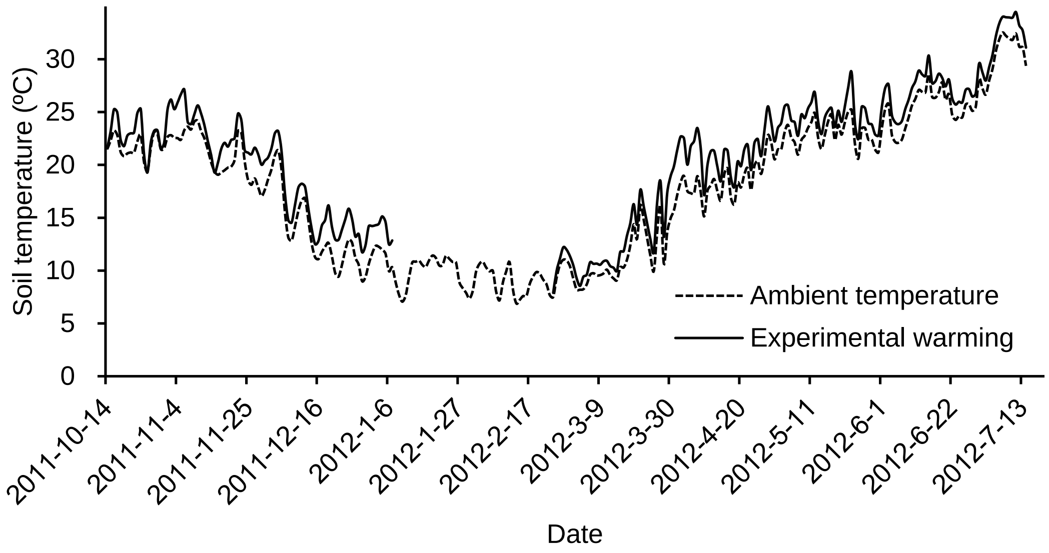

Figure S6 Soil temperature (ºC) recorded during the outdoor mesocosm experiments. Values correspond to daily average temperatures in 6 mesocosm units under ambient temperature and 12 mesocosm units exposed to experimental warming.

Table S1. Summary of three mixed-model ANCOVAs for post-hibernation body size, weight and body condition of *P. nigromaculatus* in outdoor mesocosm experiments, with experimental warming, food supply, and sex as main effects, replicates of each treatment as a random variable, age and original body size/weight/body condition as covariates.

Source of variation	Post-hibernation body size (log_{10}- transformed)		Post-hibernation weight (log_{10}- transformed)		Post-hibernation body condition	
	df	*F*	*df*	*F*	*df*	*F*
Experimental warming	2	0.817	2	4.539	2	1.935
Food supply	1	32.676***	1	196.296***	1	44.908***
Sex	1	0.299	1	3.656	1	0.014
Age	1	9.637**	1	14.810 ***	1	0.171
Original body size (log_{10}- transformed)	1	230.851***	-	-	-	-
Original weight (log_{10}- transformed)	-	-	1	350.545***	-	-
Original body condition	-	-	-	-	1	101.209***
Replicates of each treatment	2	1.599	2	0.169	2	0.343
Experimental warming × Food supply	2	0.878	2	4.436*	2	1.010
Experimental warming × Sex	1	0.308	1	2.649	1	0.572
Food supply × Sex	1	3.289	1	4.377*	1	0.130
Experimental warming × Food supply × Sex	1	0.779	1	0.625	1	0.274
Error	243		243		243	

* *P*-value < 0.05 (2-tailed); ** *P*-value < 0.01 (2-tailed); *** *P*-value < 0.001 (2-tailed).

Table S2. Summary of two mixed-model ANCOVAs for the date of entrance into and emergence from hibernation of *P. nigromaculatus* in outdoor mesocosm experiments, with experimental warming, food supply, and sex as main effects, replicates of each treatment as a random variable, age and original body condition as covariates.

Source of variation	Date of entrance into hibernation (log_{10}- transformed)		Date of emergence from hibernation (log_{10}- transformed)	
	df	*F*	*df*	*F*
Pre-hibernation warming	1	44.534***	-	-
Post-hibernation warming	-	-	1	49.985 ***
Food supply	1	0.040	1	0.009
Sex	1	0.438	1	16.888 ***
Age	1	226.413 ***	1	174.155 ***
Original body condition	1	3.508	1	0.092
Replicates of each treatment	2	1.035	2	0.173
Pre-hibernation warming × Food supply	1	0.244	-	-
Pre-hibernation warming × Sex	1	0.867	-	-
Pre-hibernation warming × Food supply × Sex	1	0.040	-	-
Food supply × Sex	1	0.615	1	0.010
Post-hibernation warming × Food supply	-	-	1	0.026
Post-hibernation warming × Sex	-	-	1	0.050
Post-hibernation warming × Food supply × Sex	-	-	1	0.220
Error	162		158	

*** *P*-value < 0.001 (2-tailed); Original body condition: determined as the residual of the regression analysis of log_{10} weight against log_{10} SVL[1], each sex calculated respectively.

1. Reading, C. J. Linking global warming to amphibian declines through its effects on female body condition and survivorship. *Oecologia* **151**, 125-131 (2007).
